# Functional Nanomaterials: From Structures to Biomedical Applications

**DOI:** 10.3390/molecules27217492

**Published:** 2022-11-03

**Authors:** Wansong Chen, Keyin Liu, Jianhua Zhang

**Affiliations:** 1 State Key Laboratory of Biobased Material and Green Papermaking, Qilu University of Technology, Shandong Academy of Sciences, Jinan 250316, China; 2 College of Chemistry and Chemical Engineering, Central South University, Changsha 410017, China; 3 Department of Polymer Science and Engineering, School of Chemical Engineering and Technology, Tianjin University, Tianjin 300072, China

In recent decades, a number of functional nanomaterials have attracted a great amount of attention and exhibited excellent performance for biomedical and pharmaceutical applications. Functional nanomaterials usually display unique physicochemical properties, nano-sized characteristics, controlled shape and versatile modification possibilities as well as well-defined multifunctionalities. A wide variety of nanomaterials, such as liposomes, polymer-based nanoparticles (NPs), carbon-based NPs, silica-based NPs, metal and metal–oxide NPs (e.g., Au, Ag and iron oxides), covalent organic frameworks and metal–organic frameworks NPs, have been developed and employed for the treatment of various diseases. These functional nanomaterials offer unprecedented opportunities for the site-specific and controllable delivery of drugs, genes, proteins and other bioactive agents. Moreover, functional nanomaterials with unique photoelectric properties can be used for photoacoustic, photothermal or photodynamic as well as hyperthermal therapy. In addition, some functional NPs can find applications for a new generation of intelligent biosensing, bioseparation, bioimaging, cell labeling and diagnosis methods as well as for monitoring cells and tissues.

Despite these tremendous advantages and great advances, clinical applications of nanomaterials as therapeutic, imaging and diagnostic agents still remain limited. For example, nanocarriers such as liposomes, micelles, dendrimers and polymeric NPs often suffer from the premature release of drugs under complex physiological conditions and uncontrollable drug release rate in vivo. From the injection site to the targeted sites, nanomedicines will be confronted with sequential drug delivery barriers, the fast clearance of blood circulation, extravasation from the blood vessels, enhanced penetration into the deep part of the nidus, effective internalization and controlled drug release inside the targeted cells. Especially for the treatment of cancer, it is necessary to develop novel and effective nanocarriers with the ability to efficiently overcome the physiological barriers for drug delivery in the body as soon as possible; especially to resolve the poor tumoral accumulation and penetration caused by the dense extracellular matrix and high interstitial fluid pressure in tumor tissues. Moreover, to overcome drug resistance, it is highly desirable to develop multifunctional nanomedicines with the combination of multiple therapeutic modalities, such as chemotherapy, photothermal therapy, photodynamic therapy, chemodynamic therapy, radiotherapy, starving therapy, and immunotherapy. Additionally, the stability and degradability as well as biosafety of most nanomedicines in biofluids should be carefully evaluated before their administration to humans. In light of the above-mentioned issues, it is imperative to develop novel and effective nanomaterials for biomedical and pharmaceutical applications.

In this Special Issue, we present original research and review articles with a focus on functional nanomaterials in biomedicine. In the direction of functional nanocarriers, Carvalho et al. reported a type of dual functionalized NPs for the synergistic delivery of levodopa and curcumin. The NPs were derived from the self-assembly of positively charged diblock copolymer NH_2_–poly(ethylene oxide)- poly(ε-caprolactone) (NH_2_–PEO–PCL) and were then modified by the addition of glutathione on the outer surface. The results indicate that the developed biodegradable NPs with blood compatibility and low cytotoxicity can pass the blood–brain barrier, target the brain tissue, and provide a more sustained release of drugs for potential application in the treatment of Parkinson’s disease [1]. Lee et al. reported on pH-responsive metal-based biopolymer NPs for tumor-specific chemotherapy. The aminated hyaluronic acid (aHA) chains were coupled with pH-responsive 2,3-dimethylmaleic anhydride (DMA). Then, the obtained aHA-DMA was electrostatically complexed with ferrous chloride tetrahydrate (FeCl_2_/4H_2_O) and doxorubicin (DOX). The produced DOX-loaded Fe-based hyaluronate nanoparticles (DOX@aHA-DMA/Fe NPs) were found to be able to improve tumor cellular uptake due to HA-mediated endocytosis for tumor cells. The nanoparticles selectively release DOX in the acidic environment of tumor cells due to ionic repulsion, which demonstrates that they can serve as promising tumor-targeting drug carriers [2]. Dong et al. summarized the advances in the tumor microenvironment (TME)-targeted nano-delivery system, which was demonstrated to be able to regulate the distribution of drugs in the body; specifically increase the concentration of drugs in the tumor site; enhance efficacy; and reduce adverse reactions, leading to a significant improvement in the effect of tumor therapy. This comprehensive review exhibited the principles and strategies of the design and utilization of the particular microenvironment of tumors to design functional NPs for the treatment and diagnosis of tumors [3].

In the direction of multimodal imaging and phototherapy of cancer and bacterial infections, Ling et al. summarized the recent progress of the general preparation and functionalization of graphene and related nanocomposites as theranostic materials. The graphene nanocomposites act as outstanding carriers for various therapeutic organic drugs or imaging probes. Moreover, graphene nanocomposites provide a robust platform for self-acting luminescent for confocal laser scanning microscopy, magnetic resonance imaging, computed tomography, positron emission tomography and photoacoustic imaging. Additionally, graphene nanocomposites can be applied in photothermal and photodynamic therapies against different cancers and bacterial infections [4]. Zhang et al. comprehensively summarized the strategies and applications of organic NPs, especially polymer-based NPs, for the delivery of iodinated contrast media in X-ray computed tomography (CT) imaging. They mainly focused on the use of polymeric nanoplatforms to prolong circulation time, reduce toxicity and enhance the targetability of iodinated contrast media. These organic NPs, such as PEGylated liposomes, nanoemulsions, micelles, polymersomes, dendrimers and natural NPs, have exhibited great potential in the development of functionalized contrast media with better biocompatibility, a longer circulation time and more efficient targeting capabilities [5].

In the direction of antibacterial, antimicrobial, antiviral and antioxidant applications of functional NPs, Tong et al. systematically summarized the preparation, characterization and regulation of single-atom nanozymes (SAzymes) through pyrolysis and defect engineering. The strategies of surface modification for SAzymes for their biomedical applications were also discussed. Due to their high atom utilization, the unsaturated coordination of active centers, and geometric structures similar to those of natural enzymes, SAzymes were demonstrated to possess tremendous potential for application in the biomedical field, especially in those of reactive oxygen species (ROS) scavenging and antibacterial therapy [6]. Zhang et al. systematically summarized the principles for the preparation and application of electrostatic spinning, especially of electrospun fibers or membranes functionalized with metal-based nanocrystals. The metal-based nanocrystal-modified nanofibers exhibited high potential in many applications, especially for antimicrobial applications [7]. Liu et al. reported that the label-free peptide substrate was able to induce the aggregation of gold nanoparticles (AuNPs) through electrostatic interactions, leading to the cleavage of the peptide by the severe acute respiratory syndrome coronavirus 2 main protease (Mpro). As a result, the visual analysis of Mpro activity according to color change of the AuNPs suspension can be achieved. Moreover, the co-assembly of AuNPs and peptides was coated on the peptide-covered electrode surface, thereby facilitating the development of a simple and sensitive electrochemical method for Mpro detection in serum samples, which was valuable for the development of effective antiviral drugs [8]. Pyrzyńska et al. investigated the influence of reaction conditions and clean-up procedure on shape, size and antioxidant activity of selenium nanoparticles (SeNPs). They found that the size and morphology of SeNPs can be controlled by the clean-up step. Moreover, the antioxidant activity often depends on the nanoparticles size and homogeneity of SeNPs [9].

In the direction of the treatment of neurogenic disease, Wang et al. comprehensively summarized the recent progress in nanomaterials-based methodologies for inhibiting amyloid-β peptides aggregation. Some nanomaterials, such as gold NPs, carbon-based NPs, transition oxide two-dimensional (2D) nanomaterials, metal–organic frameworks (MOF) and self-assembled nanomaterials were demonstrated to be able to directly interact with amyloid-β peptides to inhibit their aggregation. In addition, some nanomaterials with photosensitive properties can influence the format of amyloid-β peptides, exhibiting great potential in Alzheimer’s disease treatment [10]. In the direction of the treatment of cardiovascular disease, Wang et al. systematically summarized the advances in the preparation and utilization of cell-membrane coated nanoparticles (CMCNPs) for the treatment of cardiovascular disease. Due to their biomimetic properties, such CMCNPs can avoid immune clearance and thus prolong nanocarriers’ circulation time. Moreover, the functional proteins on the cloaked cell membranes can impart CMCNPs with additional biological properties, such as selective adherence, inflammatory site targeting and endothelium penetration. All these features significantly enhance the therapeutic efficacies of CMCNPs in treating cardiovascular diseases [11].

We hope this Special Issue provides researchers with information to help them understand the advanced strategies of functional nanoplatforms in biomedical and pharmaceutical applications, inspiring new ideas for future research directions and research activities.

## References

[1] Mogharbel B.F., Cardoso M.A., Irioda A.C., Stricker P.E.F., Slompo R.C., Appel J.M., de Oliveira N.B., Perussolo M.C., Saçaki C.S., da Rosa N.N. (2022). Biodegradable Nanoparticles Loaded with Levodopa and Curcumin for Treatment of Parkinson&rsquo′s Disease. Molecules.

[2] Bae Y., Kim Y., Lee E.S. (2021). Endosomal pH-Responsive Fe-Based Hyaluronate Nanoparticles for Doxorubicin Delivery. Molecules.

[3] Sun Y., Li Y., Shi S., Dong C. (2021). Exploiting a New Approach to Destroy the Barrier of Tumor Microenvironment: Nano-Architecture Delivery Systems. Molecules.

[4] Gollavelli G., Ghule A.V., Ling Y.-C. (2022). Multimodal Imaging and Phototherapy of Cancer and Bacterial Infection by Graphene and Related Nanocomposites. Molecules.

[5] Zhang P., Ma X., Guo R., Ye Z., Fu H., Fu N., Guo Z., Zhang J., Zhang J. (2021). Organic Nanoplatforms for Iodinated Contrast Media in CT Imaging. Molecules.

[6] Song H., Zhang M., Tong W. (2022). Single-Atom Nanozymes: Fabrication, Characterization, Surface Modification and Applications of ROS Scavenging and Antibacterial. Molecules.

[7] Li H., Xu M., Shi R., Zhang A., Zhang J. (2022). Advances in Electrostatic Spinning of Polymer Fibers Functionalized with Metal-Based Nanocrystals and Biomedical Applications. Molecules.

[8] Feng Y., Liu G., La M., Liu L. (2022). Colorimetric and Electrochemical Methods for the Detection of SARS-CoV-2 Main Protease by Peptide-Triggered Assembly of Gold Nanoparticles. Molecules.

[9] Sentkowska A., Pyrzyńska K. (2022). The Influence of Synthesis Conditions on the Antioxidant Activity of Selenium Nanoparticles. Molecules.

[10] Huang Y., Chang Y., Liu L., Wang J. (2021). Nanomaterials for Modulating the Aggregation of β-Amyloid Peptides. Molecules.

[11] Zhu C., Ma J., Ji Z., Shen J., Wang Q. (2021). Recent Advances of Cell Membrane Coated Nanoparticles in Treating Cardiovascular Disorders. Molecules.

